# ﻿Redescription of the hispidoberycid, *Hispidoberyxambagiosus* Kotlyar, 1981 from Taiwan, with comments on its morphology (Beryciformes, Stephanoberycoidei, Hispidoberycidae)

**DOI:** 10.3897/zookeys.1182.111296

**Published:** 2023-10-11

**Authors:** Yo Su, Chien-Hsiang Lin, Hsuan-Ching Ho

**Affiliations:** 1 Department of Marine Biotechnology and Resources, National Sun Yat-sen University, Kaohsiung, Taiwan National Sun Yat-sen University Kaohsiung Taiwan; 2 Biodiversity Research Center, Academia Sinica, Taipei, Taiwan Biodiversity Research Center, Academia Sinica Taipei Taiwan; 3 Department and Graduate Institute of Aquaculture, National Kaohsiung University of Science Technology, Kaohsiung, Taiwan National Kaohsiung University of Science Technology Kaohsiung Taiwan; 4 Institute of Marine Biology, National Donghwa University, Pingtung, Taiwan National Donghwa University Pingtung Taiwan; 5 Research Associate, Australian Museum, Sydney, Australia Research Associate, Australian Museum Sydney Australia

**Keywords:** biodiversity, biogeography, ichthyology, otolith, taxonomy

## Abstract

A rare spiny-scale pricklefish, *Hispidoberyxambagiosus* Kotlyar, 1981, is redescribed based on four specimens collected from Taiwan. Their sampling locality represents the northernmost record of the family, which extends the family’s distribution from the eastern Indian Ocean and the South China Sea to northeastern Taiwan in the northwestern Pacific Ocean. A detailed description of these specimens and the first description of its sagittal otoliths are provided. In addition, the specimens are compared with other known specimens. Intraspecific variation of some morphological characters are discussed.

## ﻿Introduction

The fish order Beryciformes (Nelson et al. 2016) currently comprises eight families and about 123 valid species distributed worldwide (Fricke et al. 2023). Most members are deep-sea fishes, some of which live at depths to 5308 m (Kotlyar 1996). The monotypic family Hispidoberycidae was established by Kotlyar (1981) to accommodate the new genus and new species *Hispidoberyxambagiosus* Kotlyar, 1981. The species was described based on the holotype and a non-type specimen collected from off the northwestern tip of Sumatra and the south coast of Java in the eastern Indian Ocean.

Specimens of *H.ambagiosus* appear to be extremely rare in collections worldwide, with only five specimens known from the South China Sea and East Indian Ocean (Yang et al. 1988; Kotlyar 1991, 1996, 2004). Known specimens were collected from depths of 560–1019 m, and ecology and biology of the species are still poorly known. Kotlyar (1991, 1996) described some osteological features and reviewed all available information on the family and its presumed relationships.

Recently, four specimens initially identified as *Barbourisiarufa* Parr, 1945 were found in the Pisces collection of the Biodiversity Research Center, Academia Sinica, Taipei, Taiwan (ASIZP). After a detailed examination, these specimens are re-identified as H.ambagiosus based on their unique characteristics. These specimens represent the first record of the species, genus, and family from Taiwan, as well as the third formal record in history. A detailed description of these specimens and the first description of its sagittal otoliths are provided; these specimens are also compared to the data of other known specimens.

## ﻿Materials and methods

Classification of taxonomic rank follow Nelson et al. (2016). Terminology and methodology follow Kotlyar (1996) and Su et al. (2023), with body depths measured at greatest depth and both dorsal- and anal-fin origins and body width additionally measured at lateral-line origin. Measurements of forehead length follow Su et al. (2022) and are abbreviated as HF1 and HF2. Counts of paired-fin characters and lateral-line scales were presented as left/right whenever available. Vertebral counts follow Kotlyar (1991), with the second ural centrum counted as the last vertebra. Only vertebrae with ribs are included in the counts of precaudal vertebrae. The counts of vertebrae were determined by x-radiograph. Terminology of lateral-line canals follow Jakubowski (1974) and Kotlyar (1991). In addition, terminology and description of otoliths follow Lin and Chang (2012) and Nolf (2013). The distribution map was generated from Ocean Data View (Schlitzer 2023).

Measurements were taken using 150 mm digital calipers or 300 mm calipers and rounded to the nearest 0.1 mm. Morphometric data were presented as a percentage of standard length (SL) and/or as a percentage of head length (HL), except where otherwise indicated. Specimens are deposited at Academia Sinica, Biodiversity Research Center, Taipei, Taiwan (**ASIZP**), and the Pisces Collection, National Museum of Marine Biology and Aquarium, Pingtung, Taiwan (**NMMB-P**). The sagittal otoliths of ASIZP 81665 were taken and deposited at the marine paleontology lab, Biodiversity Research Center with catalog number CHLOL 969.

## ﻿Results

### ﻿Family Hispidoberycidae Kotlyar, 1981

Chinese name: 刺金眼鯛科

#### ﻿ Hispidoberyx ambagiosus

Taxon classificationAnimaliaBeryciformesHispidoberycidae

Kotlyar, 1981

0DE7DA14-29AF-5FF3-B64B-D236A23B89D5

**Figure 1.**
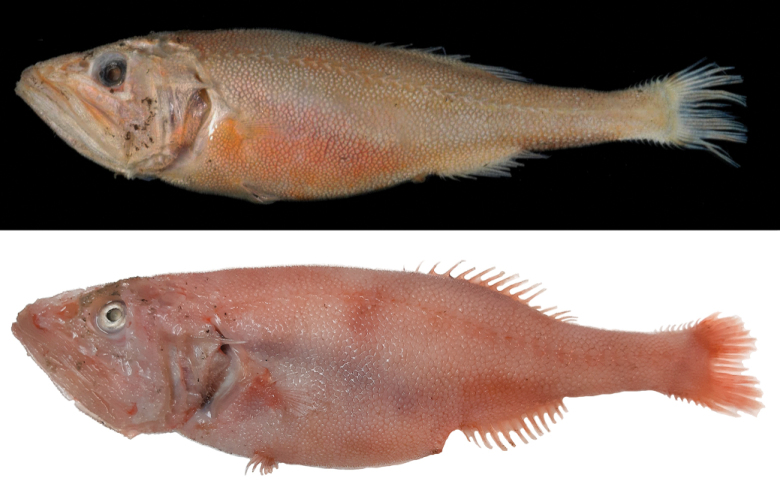
Fresh specimens of *Hispidoberyxambagiosus* Kotlyar, 1981 **A**ASIZP 64539, 154.7 mm SL (after a few months of refrigeration) **B**ASIZP 81665, 162.0 mm SL. Photographed by J.-F. Huang. Not to scale.

Figs 1 , 8 , Tables 1 , 2 English name: Spiny-scale pricklefish New Chinese name: 神秘刺金眼鯛

##### Literature records.

*Hispidoberyxambagiosus* Kotlyar, 1981: 413 (type locality: off northwestern tip of Sumatra, eastern Indian Ocean, 3°46'00"N, 95°00'00"E, depth 800–875 m. Holotype: ZMMU-P 15416): Yang et al. 1988: 3 (new record from the South China Sea). Kotlyar 1991: 100 (osteology). Kotlyar 1996: 252 (in part). Paxton in Randall and Lim 2000: 600 (listed). Kotlyar 2004: 1 (description). Kimura 2020 (phylogeny).

##### Specimens examined.

ASIZP 63512, 134.8 mm SL, bottom trawl, depth 650–800 m, 10 Jun. 1999, coll. D.-M. Chen. ASIZP 64539, 154.7 mm SL, bottom trawl, 28 Aug. 2002, coll. H.-C. Ho. ASIZP 76178, 153.5 mm SL, bottom trawl, 24 Apr. 2015, coll. M.-Y. Lee. ASIZP 81665, 162.0 mm SL, bottom trawl, 25 July 2020, coll. C.-H. Lin et al. All collected from Daxi fishing port (ca 24°53'37"N, 121°55'26"E), Yilan, northeastern Taiwan.

**Figure 2.**
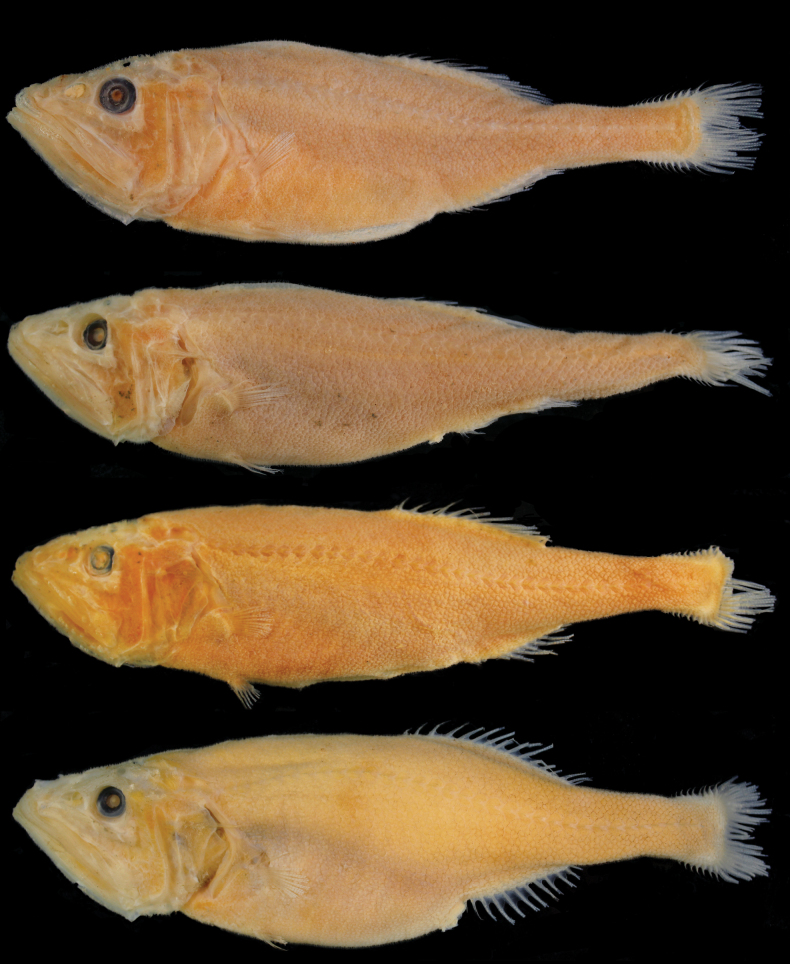
Preserved specimens of *Hispidoberyxambagiosus* Kotlyar, 1981. **A**ASIZP 63512, 134.8 mm SL**B**ASIZP 64539, 154.7 mm SL**C**ASIZP 76178, 153.5 mm SL**D**ASIZP 81665, 162.0 mm SL. Not to scale.

Otolith (a pair of sagittae): CHLOL 969, otolith length 2.2 (left) and 2.3 (right) mm, taken from ASIZP 81665.

##### Description of Taiwanese specimens.

Meristic and morphometric data are provided in Tables 1 and 2.

**Table 1.** Meristic characters of *Hispidoberyxambagiosus* Kotlyar, 1981. Data of other specimens were retrieved from Kotlyar (1981, 1996) and Yang et al. (1988). Paired characters are presented as left/right whenever available.

	This study				Kotlyar (1981)	Yang et al. (1988)	Kotlyar (1996)
	ASIZP 63512	ASIZP 64539	ASIZP 76178	ASIZP 81665	Holotype; non-type (*n* = 2)	(*n* = 2)	Holotype; non-types (*n* = 3)
Dorsal-fin elements	V, 11	V, 11	V, 11	V, 10	IV–V, 10	V, 10	IV–V, 10
Pectoral-fin elements	12/12	12/13	12/12	12/12	12	11–12	12–13
Anal-fin elements	III, 10	II, 10	III, 10	II, 10	III, 9	III, 9	II–III, 9
Pelvic-fin elements	I, 7/ I, 7	I, 7/ I, 7	I, 7/ I, 7	I, 7/ I, 7	I, 6	I, 7	I, 7
Caudal-fin elements	10+10+9+10	9+10+9+9	9+10+9+10	9+10+9+9	9+10+9+9	–	–
Gill rakers	5+1+11=17	5+1+11=17	5+1+13=19	4+1+10=15	5–6+1+12=18–19	6+1+9–11=16–18	5–6+1+9–12=15–19
Pseudobranchial filaments	11	11	10	10	–	–	–
Lateral-line scale	34/34	33/34	36/36	34/33	32	33–34	32–34
Scale rows above lateral line	16	15	15	18	–	–	–
Scale rows below lateral line	30	27	31	28	–	–	–
Vertebrae	13+23=36	13+23=36	13+23=36	13+23=36	12+22=34	–	12–13+22=34–35

**Table 2.** Morphometric characters of *Hispidoberyxambagiosus* Kotlyar, 1981. Data of other specimens were retrieved from Kotlyar (1981, 1996) and Yang et al. (1988). Abbreviations: A, Anal-fin; C, Caudal-fin; D, Dorsal-fin; H, head length; HF, forehead height; P, Pectoral-fin; SL, standard length; V, Pelvic-fin.

	This study				Kotlyar (1981)	Yang et al. (1988)	Kotlyar (1996)
	ASIZP 63512	ASIZP 64539	ASIZP 76178	ASIZP 81665	Holotype; Non-type (*n* = 2)	*n* = 2	Holotype; Non-types (*n* = 3)
SL (mm)	134.8	154.7	153.5	162.0	162–181	173–175	156–181
%SL							
HL	31.3	30.6	31.3	31.3	29.6–33.1	31.4–31.7	27.6–33.1
Head depth	22.8	21.9	21.3	21.6	–	–	21.0–22.1
Body width	9.1	11.0	10.2	11.1	–	–	–
Predorsal length	53.5	51.0	52.9	53.4	53.8–55.1	52.0–56.0	51.8–55.8
Prepectoral length	33.9	33.7	31.9	35.1	32.7–36.5	–	32.7–36.5
Prepelvic length	36.0	37.2	35.6	37.4	34.1–38.6	36.0–36.9	34.6–39.1
Preanal length	64.4	65.2	65.3	63.7	61.0–66.3	62.4–63.4	61.0–66.3
Snout length	9.6	9.4	8.9	9.6	12.2–12.3	12.1–12.7	11.2–12.3
Eye diameter	6.5	5.9	5.4	5.7	4.3–4.4	4.5–4.6	4.3–4.8
Interorbital width	11.4	10.5	11.0	10.9	–	9.8–19.0	9.0–11.0
Upper-jaw length	21.4	20.8	20.2	21.1	20.3–22.1	19.0–20.0	19.9–22.1
Lower-jaw length	23.2	22.8	21.5	23.1	22.2–24.9	–	21.5–24.9
HF1	2.0	2.1	1.9	1.5	–	–	1.6–4.3
HF2	5.9	5.2	5.6	5.4	–	–	–
Postorbital length	14.7	14.0	14.5	14.3	12.3–13.3	–	12.1–13.3
D–P length	27.9	23.3	25.9	26.6	–	–	–
D–V length	32.2	29.3	32.4	34.5	–	–	–
Body depth at D origin	27.7	22.1	23.4	28.1	–	–	–
Body depth at A origin	21.0	18.1	20.4	22.4	–	–	–
Greatest body depth	29.7	25.7	25.4	29.2	24.1–29.3	–	24.1–29.3
V spine	6.7	5.8	broken	6.2	–	–	–
P–V length	5.4	6.8	6.2	7.4	4.9–6.1	–	4.9–6.7
D–A length	23.1	22.0	23.8	24.2	–	–	–
V–A length	29.6	31.3	32.1	29.3	22.8–27.2	–	27.2–29.8
D length	22.1	22.6	22.3	24.0	21.6–22.1	–	21.6–22.1
First D spine	3.4	2.5	broken	2.4	–	–	–
Second D spine	4.6	4.4	3.3	3.1	–	–	–
Last D spine	7.6	6.1	5.2	broken	–	–	–
A length	15.4	13.8	14.3	15.4	12.3–13.8	–	12.3–13.8
Last A spine	broken	broken	broken	4.4	–	–	–
Postanal length	23.8	23.2	23.6	23.2	23.2–24.7	–	–
Postdorsal length	25.1	25.8	24.3	23.4	26.0–27.8	–	–
Caudal-peduncle height	8.2	8.3	7.7	8.1	8.0–8.3	8.5–8.6	8.0–8.3
longest gill raker	4.7	4.1	4.6	5.0	4.0–4.4	–	4.0–4.4
gill filaments at angle	2.0	2.0	2.1	1.5	–	–	–

**Figure 3.**
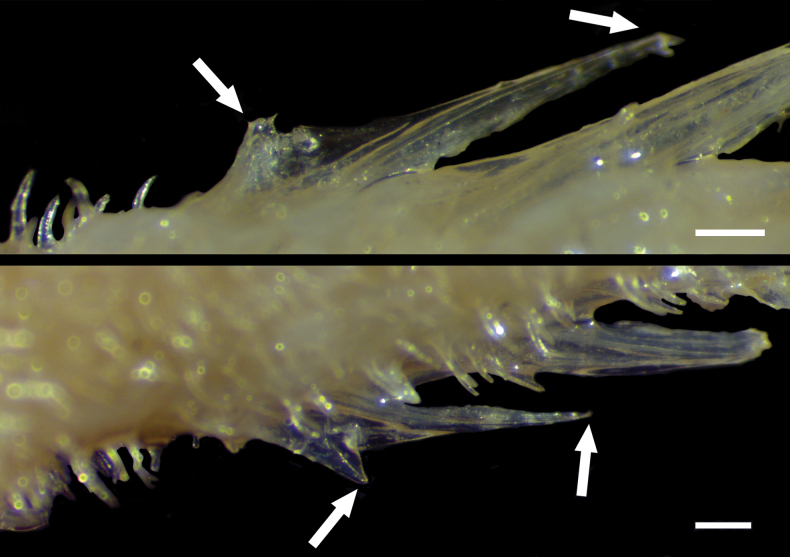
Close-up images of *Hispidoberyxambagiosus* Kotlyar, 1981, ASIZP 63512, 134.8 mm SL, featuring the fusion of the first two spines on (**A**) dorsal and (**B**) and anal fins (tips indicated by arrows). Scale bar: 500 μm.

**Figure 4.**
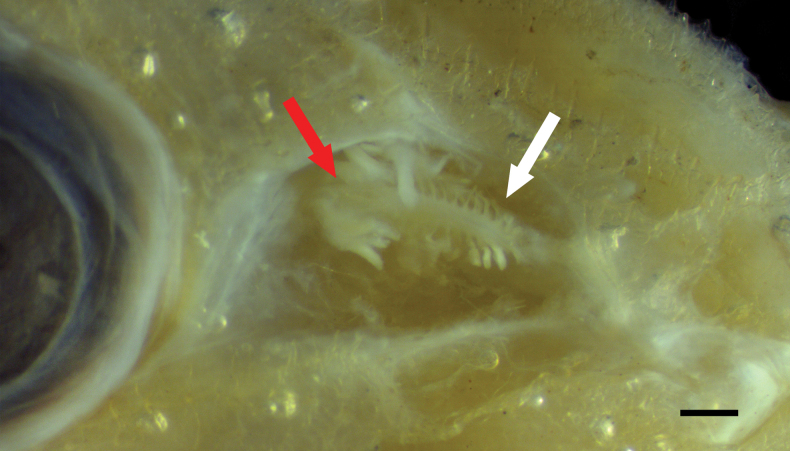
Close-up image of *Hispidoberyxambagiosus* Kotlyar, 1981, ASIZP 63512, 134.8 mm SL, featuring the nasal organ (white arrow) in the right olfactory chamber. Red arrow indicates the position of Tominaga’s organ (beneath and behind nasal organ). Anterior to right. Scale bar: 1 mm.

Dorsal-fin elements V, 10–11, first 2 spines fused in 2 specimens (Fig. 3A). Pectoral-fin elements 12/12–13, uppermost 2 and lowermost 1 or 2 rays unbranched. Pelvic-fin elements I, 7/I, 7. Anal fin-elements II–III, 10, first 2 spines fused in all specimens (Fig. 4B; 1 specimen unavailable). Principal caudal-fin rays 10 + 9, uppermost and lowermost rays unbranched; procurrent caudal-fin rays 9–10 on both upper and lower lobes. Gill rakers on outer face of first arch 4–5 + 1 + 10–13 = 15–19 (total). Pseudobranchial filaments 10–11. Lateral-line scales 33–36/33–36; scale rows above lateral line 15–18; scale rows below lateral line 27–31. Vertebrae 13 + 23 = 36; branchiostegal rays 8.

Body slender for stephanoberycoid, greatest depth 3.4‒3.9 in SL, depth at dorsal- and anal-fin origins 3.6‒4.5 and 4.5‒5.5 in SL, respectively; body laterally compressed and oval in trunk section, its width 4.4‒4.7 in SL. Head somewhat oval, length 3.2‒3.3 in SL; its height 1.4‒1.5 in HL; upper profile of head nearly straight, gently curved to dorsal-fin origin; forehead flat, HF1 14.3‒20.3 and HF2 5.3‒5.9 in HL; eye diameter 4.8‒5.8 in HL; tip of snout slightly rounded, not extending before premaxilla, its length 3.3‒3.5 in HL; interorbital width 2.8‒2.9 in HL.

Mouth oblique, upper-jaw length 1.5 in HL; posterior end of maxilla rounded, reaching vertical through posterior margin of eye; lower jaw slightly larger than upper jaw and protruding before upper jaw, length 1.3‒1.5 in HL. Two nostrils at same horizontal through center of eye; both nostrils rounded, slightly oval, with posterior nostril much larger than anterior one; both nostrils immediately in front of eye. Tominaga’s organ (Fig. 4; *sensu*Paxton et al. 2001) present in olfactory chamber, mostly embedded behind nasal organ (Fig. 4). Nasal organ large and oval, bearing leaf-like appendages.

Symphysis of premaxillae notched and edentate. Symphysis of dentaries slightly notched and edentate. Supramaxilla single, with long needle-like process extending anteriorly and rectangular process posteriorly; covering about half of posterior portion of maxilla.

**Figure 5.**
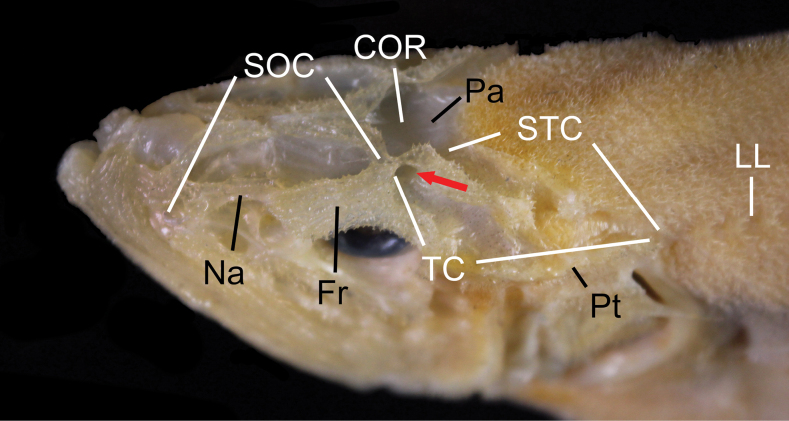
Dorsal-lateral view of *Hispidoberyxambagiosus* Kotlyar, 1981, ASIZP 81665, 162.0 mm SL, showing sensory canals (white) on head and nearby bones (black). Red arrow indicates the fenestration connecting COR and TC. Abbreviations: COR, coronal commissure; Fr, frontal; LL, lateral line; Na, nasal; Pa, parietal; Pt, posttemporal; SOC, supraorbital canal; STC, supratemporal canal; TC, temporal canal. Anterior to left. Not to scale.

Bony ridges associated with skeletons of head, jaws, snout, and operculum covered with small spinules. Bony ridges on head forming sensory canals (Fig. 5); supraorbital canal running from nasal, frontal, connected to coronal commissure at parietal bones, and divided into temporal and supratemporal canal on posttemporal bone, and joined together, connected to lateral line. Fenestration present on frontal bone connecting coronal commissure and temporal canal (Fig. 5; red arrow). Opercle with 1 strong central spine. Posttemporal bone without spine. Pectoral girdle smooth, without any spines. Premaxilla with villiform teeth, its outer surface completely exposed and bearing 2 or 3 ridges anteriorly on its ascending process; its end extending to posterior end of maxilla. Dentary with villiform teeth on its medial face. Palatine and vomer with villiform teeth.

Gill rakers rod-shaped, laterally compressed, their inner surfaces covered with small teeth; rakers on outer row of first arch longer than remainder, longest gill raker shorter than eye diameter; small bump-like rakers on inner surfaces of outer 3 arches; outer-row rakers gradually shorter from first to fourth arch, with very short rakers on outer row of fourth arch; no tooth patches present between rakers on all 4 arches. Narrow, villiform tooth patch present on fifth ceratobranchial. Long, oval tooth patch on third epibranchial arch. Large, teardrop-like villiform tooth patch on third pharyngobranchial. Small, rounded villiform tooth patch on fourth pharyngobranchial. Gill filaments on first arch short, about 1/3–1/2 length of longest opposite rakers. Pseudobranch present and short.

**Figure 6.**
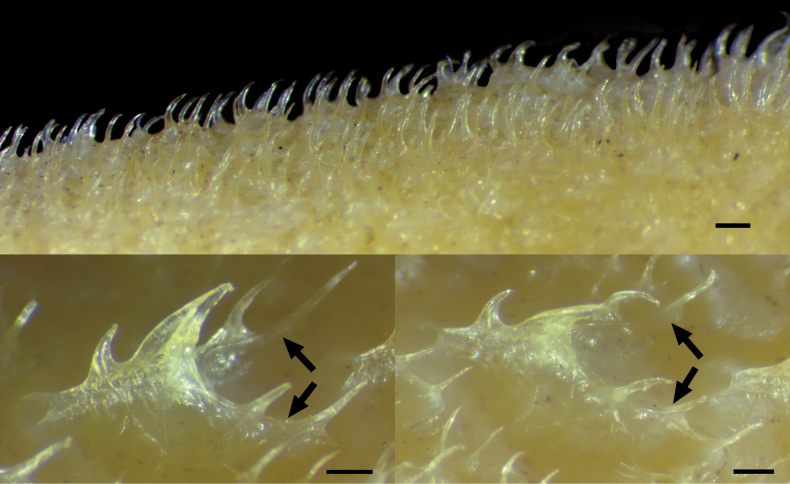
Body and lateral-line scales of *Hispidoberyxambagiosus* Kotlyar, 1981, ASIZP 81665, 162.0 mm SL. **A** body scales on nape **B** lateral-line scales on anterior portion **C** lateral-line scales on posterior portion. Anterior to left. Scale bars: 500 μm.

Prickle-like body scales adherent (Fig. 6A), covering entire body, operculum, and cheeks; spinules on body scales needle-like and curved backwards, their numbers variable: scales on nape with ca 2‒7 spinules; scales on abdominal region with 2‒7 spinules; scales on dorsum with 2‒14 spinules; scales above anal-fin base with 4‒11 spinules; scales on caudal peduncle with 3‒16 spinules. Lateral-line scales shield shaped (Fig. 6B, C) with 2 posterior branches, each bearing 1‒3 (modally 2) spines curving backwards; center of each scale with 2 or 3 (rarely 1) central spines curving and pointing backwards; all lateral-line scales distinctly larger than body scales; lateral-line canals opened at both anterior and posterior ends of scales. No scutes on abdominal region. No scales on gular region and isthmus. Predorsal scales not enlarged and not aligned in straight line.

Dorsal fin low, situated posteriorly, slightly anterior to anal-fin origin. Origin of pectoral fin situated lower than horizontal through ventral margin of eye. Origin of pelvic fin below and slightly behind pectoral-fin base. Both pectoral and pelvic fins short, their tips clearly anterior to vertical through anal-fin origin. Anal-fin base rather short, its end at same vertical through end of dorsal-fin base. Caudal fin moderately small, slightly forked. All fin rays fragile and possess spinules on lateral surfaces, except for procurrent caudal-fin rays (sometimes also absent on anterior most dorsal- and anal-fin spines).

Lateral line single, originating behind and slightly lower than posterior tip of posttemporal bone; its anterior portion slightly curved and raised, with downturn below dorsal-fin base, and nearly straight posterior portion; its end anterior to caudal-fin base. Anus situated immediately anterior to anal-fin origin. Caudal peduncle stout, length 1.3 in HL, height 3.7‒4.1 in HL. Light organs absent. No trace of swim bladder.

##### Otoliths.

**Figure 7.**
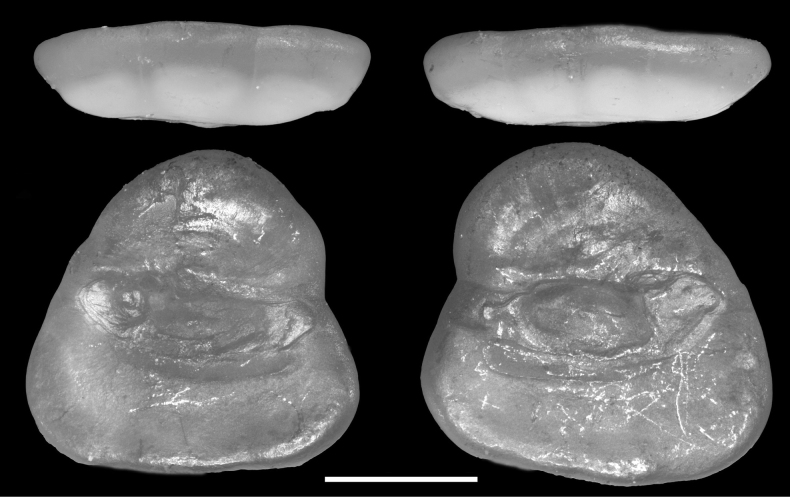
Otoliths (a pair of sagittae) of *Hispidoberyxambagiosus* Kotlyar, 1981. Specimens (CHLOL 969) were taken from ASIZP 81665 **A, B** left otolith, 2.2 mm otolith length **C, D** right otolith, 2.3 mm otolith length **A, C** ventral views **B, D** inner (mesial) views. Scale bar: 1 mm.

(Fig. 7). Otoltihs triangular, with horizontal, long ventral rim, oblique posterior and anterior rims, and short but rounded dorsal rim. Slightly notched in anterior rim, forming brief but obtuse rostrum and antirostrum. All margins smooth. Otoliths notably thickened, with inner and outer faces nearly flat. Sulcus centrally positioned, not divided into ostium and cauda, open anteriorly, slightly bent upward posteriorly but not reaching posterior rim. Cristae not well delineated. Single, large colliculum centrally located, but shape of its posterior margin varies greatly; largely extended posteriorly in right otolith, but deeply indented in left one.

##### Coloration.

When fresh (Fig. 1), entire body, including head, fin rays, and fin membranes uniformly pinkish to reddish. When preserved (Fig. 2), body uniformly pale, including entire oral cavity, gill rakers, inner face of operculum, stomach, and intestine. Membrane of kidney and ventral side of peritoneum scattered with pepper-like black pigments. Pelvic fin slightly dusky, while other fins pale.

##### Size.

This is a moderately small species of stephanoberycoid, attaining at least 181 mm SL (holotype; Kotlyar 1981). Our largest specimen (ASIZP 81665; 162.0 mm SL) is a mature female with developing eggs, suggesting that it may mature at this size.

## ﻿Discussion

### ﻿Distribution

**Figure 8.**
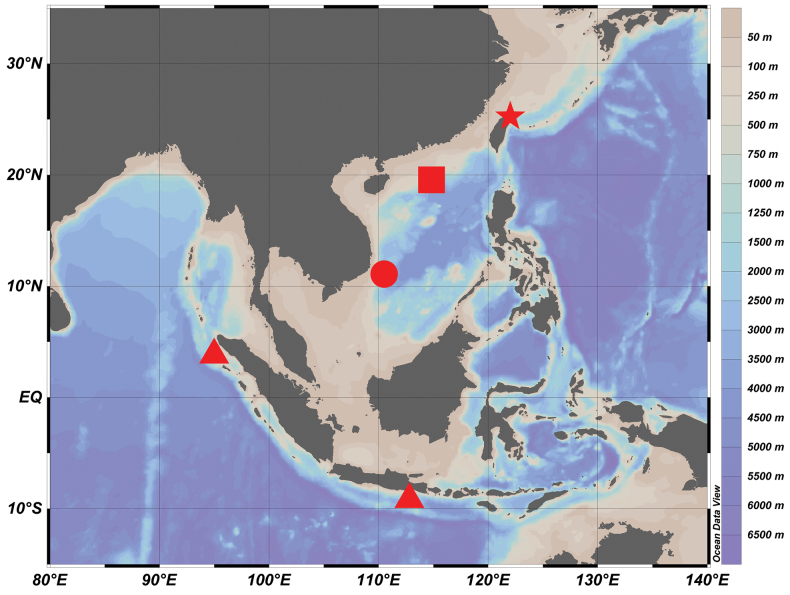
Distribution map of *Hispidoberyxambagiosus* Kotlyar, 1981. Data source: star = this study; triangle = Kotlyar (1981); square = Yang et al. (1988); circle = Kotlyar (1991).

*Hispidoberyxambagiosus* was originally described from the eastern Indian Ocean (Kotlyar 1981) and subsequently recorded from the South China Sea (Yang et al. 1988; Kotlyar 1991). Our specimens represent the northernmost record of this species, suggesting a wide, but more or less restricted distribution in the western Pacific and eastern Indian Ocean; the known bathymetric range is 560‒1019 m (Yang et al. 1988; Kotlyar 1991). With the new information presented here, the geographic range this species is now known to extend from the South China Sea to northeastern Taiwan, northwestern Pacific Ocean (Fig. 8).

### ﻿Fin elements

The counts of fin rays of our specimens generally agree with those of Kotlyar (1996), with the exception that some of our specimens have one more dorsal-fin soft ray (10‒11 vs 10 in Yang et al. 1988; Kotlyar 1996; Table 1) and consistently more anal-fin soft rays (10 vs 9). Notably, two of our specimens have their first two dorsal-fin spines and three specimens have their first two anal-fin spines fused as a single, double-tipped spine: we counted them as a single spine. Although Kotlyar (1981, 1991) did not mention such conditions, our specimens have the same number of fin spines (IV–V and II–III in dorsal and anal fins, respectively; Kotlyar 1981, 1996). Moreover, we found that all fin soft rays of our specimens are rather delicate, hindering precise measurements of them. These unique observations were not documented in previous works (Kotlyar 1981, 1991, 1996; Yang et al. 1988).

### ﻿Lateral-line scales

The number of lateral-line scales generally agrees with the data provided by Kotlyar (1996), with the exception that one of our specimens has 36 lateral-line scales (vs 32–34 in Kotlyar 1996; Table 1). On the other hand, the overall shape of the lateral-line scales generally agrees with Kotlyar (1981: fig. 3). However, our specimens have longer posterior branches (Fig. 6B, C) and usually bear two spines (vs only 1 spine in all 3 scales, as illustrated by Kotlyar 1981: fig. 3). Additionally, we found all of the lateral-line scales opened at both anterior and posterior ends.

### ﻿Body scales

All body scales of *H.ambagiosus* possess long, needle-like, recurved spinules on their surfaces. The numbers of those spinules are variable, however those on the anterior and ventral sides of the body tend to have fewer spinules. Moreover, we counted 3–8, 5–8, 4–10, and 7–16 spinules on caudal-peduncle scales in the 134.8, 153.5, 154.7, and 162.0 mm SL specimens, respectively, and similar phenomena were observed in scales above the anal-fin base, on the nape, and on scales of the dorsum. Therefore, we suggest that the number of spinules on these body scales slightly increases with body size.

### ﻿Vertebrae

Because of the thickened body scales, it is difficult to determine the position of the first haemal spine. Therefore, we followed Kotlyar (1991) to include the vertebrae with pleural ribs as precaudal vertebrae and the remaining as caudal vertebrae. However, our specimens possess one additional caudal vertebra compared to previous works (23 vs 22 in Kotlyar 1981, 1991, 1996; Table 1). Although Kotlyar reported 22 caudal vertebrae in his original description, he subsequently (Kotlyar 1991) stated that the second ural centrum was not included in the original description. Nonetheless, Kotlyar (1991, 1996) provided the same number of caudal vertebrae (22) as in the original description, which may indicate that the number was not revised and caused this discrepancy in counting vertebrae numbers.

### ﻿Tominaga’s organ

The Tominaga’s organ was first described as a structure with unknown function situated between the nasal rosette and the eye in *Rondeletialoricata* Goode & Bean, 1895 by Tominaga (1970) (Paxton et al. 2001). Later, Paxton et al. (2001) found this organ only exists in three species of Stephanoberycoidei, namely *Rondeletiabicolor* Abe & Hotta, 1963, *R.loricata*, and *Gibberichthyspumilus* Parr, 1933, and these authors proposed that Rondeletiidae and Gibberichthyidae are closely related. They also provided detailed descriptions and comparisons of this organ in the three species. Moreover, they suggested that the function of Tominaga’s organ may be secretory (Paxton et al. 2001).

In this study, we confirm that Tominaga’s organ is present in *H.ambagiosus* (Fig. 4). The nasal organ is visible when the nasal membrane is removed, and the overall shape is similar to those in Rondeletiidae and Gibberichthyidae (Paxton et al. 2001); as the Tominaga’s organ lies beneath the skin behind the nasal organ, dissection is needed. Additionally, although not mentioned in the previous work (Ho et al. 2023), Tominaga’s organ is also confirmed in *Gibberichthyslatifrons* (Thorp, 1969).

### ﻿Otoliths

In this study, the sagittal otoliths of *H.ambagiosus* have been both described and depicted for the first time (Fig. 7). Notably, their peculiar shape and highly specific sulcus configuration, characterized by a singular substantial colliculum, exhibit resemblances to features observed in otoliths of Rondeletiidae and Barbourisiidae (Rivaton and Bourret 1999; Nolf 2013). A particularly striking similarity is found with the otoliths of Cetomimidae (Fitch 1979). Noteworthy parallels can be drawn between the otoliths of *H.ambagiosus* and those of *Cetomimus*, *Ditropichthys*, and *Gyrinomimus* as illustrated by Fitch (1979). These include a triangular outline with an angled dorsal rim and an elongated ventral rim, the presence of a single substantial colliculum, and less prominently developed cristae. These shared features suggest a close relationship among Stephanoberycoidei.

### ﻿Morphological variations

Variations in morphometric data of our specimens compared with those recorded by Kotlyar (1981, 1996) and Yang et al (1988) were observed. Compared with Kotlyar (1981 and 1996), our specimens have a longer eye diameter (5.4‒6.5% SL vs 4.3‒4.8% SL in Kotlyar 1996; Table 2); longer postorbital length (14.0‒14.7% SL vs 12.1‒13.3% SL); slightly longer pelvic-fin‒anal-fin length (29.6‒32.1% SL vs 27.2‒29.8% SL); slightly longer dorsal-fin length (22.1‒24.0% SL vs 21.6‒22.1% SL); slightly longer anal-fin length (13.8‒15.4% SL vs 12.3‒13.8% SL); slightly longer longest gill-raker length (4.1‒5.0% SL vs 4.0‒4.4% SL); and a shorter postdorsal length (23.4‒25.1% SL vs 26.0‒27.8% SL in Kotlyar 1981). Since most of our specimens are smaller than specimens previously recorded (134.8‒162.0 mm vs 156–181 mm in Yang et al. 1988; Kotlyar 1996), all morphometric differences we found are considered intraspecific variations.

Additionally, we suggest that the difference in snout length (8.9‒9.6% SL vs 11.2‒12.3 in Kotlyar 1996; Table 2) may be attributed to the difference in measuring landmarks. The anterior portion of the premaxilla protrudes before the snout, and thus we measured the snout length from the anterior tip of the lachrymal to the anterior margin of the eye only. It is very likely that both Kotlyar (1981, 1996) and Yang et al. (1988) included the premaxilla in their measurements of snout length, which, therefore, caused this discrepancy.

### ﻿Record of *Barbourisiarufa* from Taiwan

The studied specimens were initially identified as *Barbourisiarufa*, with this species and *H.ambagiosus* both sharing a bright-red body coloration when fresh, and a rather big mouth with the posterior end of the maxilla exceeding a vertical through the posterior margin of the eye. However, *H.ambagiosus* is readily distinguished from *B.rufa* in having the pelvic fins anteriorly situated (vs posteriorly situated at the middle of trunk in *B.rufa*; Parr 1945), presence of dorsal- and anal-fin spines (vs fin spines absent on both fins), gill chamber and peritoneum pale (vs black), and opercle with single, strong central spine (vs opercle without spines).

Although the specimens reported here as *H.ambagiosus* were the basis for the inclusion of *B.rufa* in the Taiwanese fauna (Shao 2023), another *B.rufa* specimen (ASIZP 57678), previously considered lost (S.-P. Huang pers. comm.), was relocated in the National Museum of Marine Science and Technology, Keelung Taiwan (NMMST) for exhibition (J.-F. Huang pers. comm.), and we identify that specimen here as *B.rufa*. Therefore, *B.rufa* is retained in the ichthyofauna of Taiwan.

### ﻿Comparative materials

*Barbourisiarufa*: ASIZP 57678, 312 mm SL, Bashi Channel, 21°30'00"N, 120°47'59.99"E, depth 300–400 m, 20 Jan. 1991, bottom trawl, coll. J.-W. Chen. *Gibberichthyslatifrons*: NMMB-P37435, 100.7 mm SL, off Dong-gang fishing port (ca 22°22'22"N, 120°27'34"E), Pingtung, southwestern Taiwan, 26 Dec. 2022, bottom trawl, coll. K.-H. Wu.

## Supplementary Material

XML Treatment for Hispidoberyx ambagiosus

## ﻿Additional information

### Author ORCIDs

Yo Su https://orcid.org/0000-0002-3576-9229

Chien-Hsiang Lin https://orcid.org/0000-0002-9843-9729

Hsuan-Ching Ho https://orcid.org/0000-0003-1154-601X

### Data availability

All of the data that support the findings of this study are available in the main text.
